# Utilization of Participatory Research Theory and a National Framework to Advance a State Food Security Research Agenda: A Mixed-Methods Study

**DOI:** 10.3390/ijerph21121677

**Published:** 2024-12-17

**Authors:** Barbara Gordon, Jenifer Reader, Benjamin G. Larsen, Kelsey Cooper

**Affiliations:** 1 Department of Nutrition and Dietetics, Idaho State University, Meridian, ID 83642, USA; 2 Department of Nutrition and Dietetics, Idaho State University, Pocatello, ID 83209, USA; jeniferreader@isu.edu; 3 Idaho Policy Institute, Boise State University, Boise, ID 83725, USA; larsen.be@northeastern.edu; 4 Idaho Foodbank, Meridian, ID 83642, USA

**Keywords:** food security, community-based participatory research, health disparities, research methods, nutrition, USA

## Abstract

The 2022 White House National Strategy on Hunger, Nutrition, and Health outlined goals for ending hunger in the US. Actions fell into five areas, called pillars; the goal of Pillar 5 was to enhance nutrition and food security research. This study leveraged participatory research theory and the National Strategy for developing a statewide, evidence-informed food security research agenda. A mixed-methods study employing the Community Engagement in Research Continuum (CEnR) was implemented. Engagement strategies included collecting baseline data via a statewide survey and encouraging participants of a statewide Summit to participate in a collective prioritization process. Surveys were emailed to a purposive sample of 1575 contacts; 31 registered for the in-person session. A total of 197 records were included in the survey analysis; all 31 registrants attended the in-person session. The research agenda was to include two objectives for implementation over the next two years. Academic and nonacademic stakeholders interacted as peers during the prioritization exercise. The first research objective was to conduct food assistance usage gap analyses; the second was to investigate barriers to collaboration among Idaho’s food security programs. The CEnR proved efficacious for producing a stakeholder-generated research agenda. Leveraging a national framework helped facilitate asset-based, actionable strategies within a proposed timeline.

## 1. Introduction

The 2022 White House National Strategy on Hunger, Nutrition, and Health outlined goals for ending hunger in the US [1]. Actions fell into five areas: Pillar 1—improving food access/affordability, Pillar 2—integrating nutrition and health, Pillar 3—empowering consumers to make/access healthy choices, Pillar 4—supporting physical activity, and Pillar 5—enhancing nutrition and food security research. In addition to federal initiatives, the National Strategy included a call to action for state agencies and community groups [1]. The Idaho Hunger Relief Task Force (IHRTF) embraced that call to action.

IHRTF is a nonprofit organization mobilizing statewide, private, and public resources to help mitigate food insecurity among Idahoans [2]. In 2022, 11.4% of Idahoans were food insecure [3]. Nearly double the number of Idahoans with Hispanic/Latino ethnicity were food insecure compared with the general state population, 15% vs. 8%, respectively. Four rural counties—Washington, Butte, Lewis, and Shoshone—had the highest prevalence, 15.5%, 16.4%, 16.4%, and 16.5%, respectively [3]. Children disproportionately experienced hunger. In 2022, 14.5% of Idaho children were food insecure [3]. Rates were higher among children in four rural counties: Custer, 21.4%; Butte, 22.7%; Shoshone, 23.1%; and Lewis, 24.5% [3].

Biennially, the IHRTF sponsors a Summit on Hunger and Food Security, which gathers representatives from advocacy groups, businesses, institutions of higher education, charitable emergency food providers, healthcare companies, faith organizations, rural, suburban, and urban communities, and state and local government agencies from across the state to discuss hunger among Idahoans [2]. The 2023 Hunger Summit, held in Boise, Idaho, applied the National Strategy to Idaho; 175 individuals attended this one-day meeting [4]. The agenda mirrored the organization of the National Strategy; there was a meeting track for each of the pillars. The Pillar 5 track focused on advancing food security research in Idaho [4].

Fanzo et al. note the essentiality of research in guiding a positive and sustainable path for food security [5]. Published examples of food security research agendas for individual U.S. states, however, are sparse. A 2022 Robert Wood Johnson Foundation report offered suggestions on developing a research agenda for the state of New Jersey; specifically, to employ participatory research methods and adopt an asset-based framework [6]. Davis et al. detailed the advantages of a community-university partnership for yielding relevant research queries [7]. An effective community-university partnership requires employing participatory research to engage representatives of communities affected by the issue in a systematic inquiry process [6,7,8,9,10,11,12]. The IHRTF Hunger Summit offered access to stakeholders with disparate perspectives on the potential scope of a food security research agenda.

In this study, a community-university research team leveraged participatory research theory and the National Strategy to generate a statewide, evidence-informed, asset-based food security research agenda with an actionable two-year timeframe.

## 2. Materials and Methods

This study used a version of the International Association for Public Participation (IAP2) Spectrum of Participation called the Continuum of Community Engagement in Research [13]. Principles of the community-engaged research (CEnR) continuum and participatory research theory provided a framework for data collection and analysis. Data were collected from a statewide group of stakeholders through an online survey combined with facilitated discussions at a public forum. The Idaho State University Institutional Review Board deemed this study exempt [14]. The Good Reporting of a Mixed Methods Study (GRAMMS) framework was applied in this paper (see Table S1) [15].

### 2.1. CEnR Continuum

**Figure 1:**
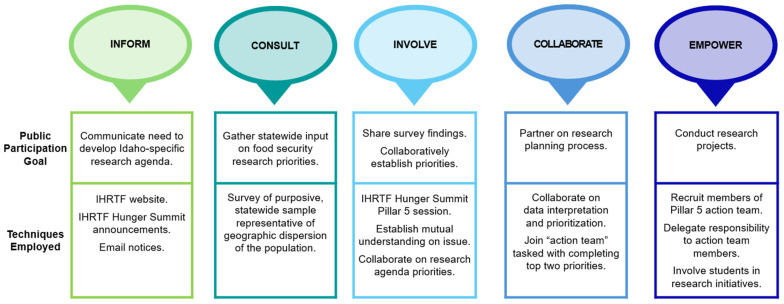
CEnR continuum yielding Idaho-specific food security research agenda.

The CEnR continuum includes five domains associated with public participation goals [13,16]. A mixed-methods approach involving all five domains was employed to ensure community members and researchers were integral and equitable team members [9]. Engagement strategies included (1) collecting baseline statewide quantitative survey data and (2) collecting qualitative data by facilitating discussions among Hunger Summit participants as part of a collective prioritization process to determine a statewide food security research agenda. The survey findings offered insights from a more diverse stakeholder group and helped ensure that all research team members possessed the same level of understanding about those perspectives. The findings of the in-depth facilitated discussions about precipitating survey themes were essential for prioritizing a collaboratively developed research agenda. Figure 1 illustrates the five domains of the CEnR continuum utilized for this study; a description follows.

Domain 1: Inform. The first degree of engagement was to “inform” stakeholders. The goal was to communicate with Idahoans about food security and the need for an Idaho-specific food security research agenda. The USDA definitions for food security and insecurity were employed [17]. Techniques included posts on the IHRTF website, inclusion of information in Hunger Summit announcements, email notices to organizational mailing list members, and personal contacts via a snowball approach. Thirty-one participants registered for the Hunger Summit Pillar 5 track.

Domain 2: Consult. The next level of engagement—consult—was implemented by gathering input via a 20-question survey (online Qualtrics platform with Transport Layer Security encryption or https). No identifying information was asked as part of the survey. Respondents were automatically assigned a study ID. If they wanted to participate in a gift card raffle incentive, participants could provide an email address. Drawing on the National Strategy, questions covered food security screening, contributing factors, and impact on nutrition/health outcomes, as well as strategies to address food insecurity (see Supplemental Figure S1).

Steps in the survey development and testing process included crafting the questionnaire, ensuring content validity via expert review, conducting additional validating testing through pilot-testing, executing the pilot-test via the online platform, and incorporating the feedback from the pilot-testers into the final survey. The expert reviewers included subject matter experts, academics, and professionals; the group included individuals familiar with survey design, data collection, and data analysis. Pilot-testers included stakeholders with various perspectives on food security. Of note, based on the pilot test, the survey was anticipated to take about 10 min to complete.

A purposive sample of 1575 contacts reflective of Idaho’s population by county was compiled. IHRTF disseminated the survey via email (3 attempts across 7.5 wks, August–September 2023). The survey link was also accessible via IHRTF’s website, and staff and volunteers sent the link via personal communications.

Domain 3: Involve. A facilitated dialogue guide was developed for the Hunger Summit Pillar 5 session. The guide included key points regarding the objective of the discussion, general ground rules to encourage respectful engagement, questions to guide the lunchtime data collection exercise and the small group discussions, and information on the reporting process. The guide went through expert review for content validity, and feedback was incorporated.

At the Hunger Summit, 31 participants (public health professionals, Idaho Foodbank staff, Tribal representatives, academics, and college students) attended the in-person, 3.5 h Pillar 5 track. A mutual level of understanding was fostered by providing an overview of the survey process and findings; a facilitated discussion followed. Participants were given a Lunchtime Networking Assignment to share what they learned and ask colleagues to identify one research agenda priority. They then shared that information with the other Pillar 5 participants and a list of themes was compiled. Participants then collaboratively created research objectives by working in small groups; they were tasked with identifying two research questions and, for each question, potential research methods, and project deliverables. An academic stakeholder was paired with each group to clarify the components of a research question, examples of research methodologies, and types of deliverables. The full group reconvened, and the small groups presented their priorities. The priorities were debated, and the full group worked together to reach an agreement on which two priorities to move forward as the statewide research agenda.

Domain 4: Collaborate. The IHRTF Hunger Summit model engaged a core team of session planners and participants committed to implementing the research agenda over the next two years. At the end of the session, participants were invited to be part of the “action team” tasked with designing and executing studies investigating the identified priorities. Academic and community volunteers are now partnering in the research process with equal involvement in the decision-making process.

Domain 5: Empower. Two research projects were completed by emerging scholars in a master of public administration program as a result of this process of participatory research. Guided by members of the action team, in May 2024, graduate students completed a literature review and in-depth interviews gathering data on the difference between the urban and rural food insecurity experience. In December 2024, another team of students completed a project studying the coalition of stakeholders with the potential of expanding produce prescription programs in Idaho.

### 2.2. Data Analysis

**Figure 2:**
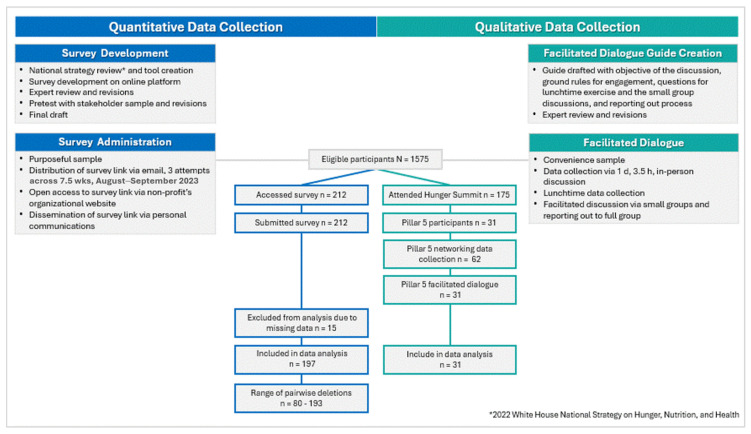
Diagram of quantitative and qualitative data collection protocols.

This study utilized a mixed-methods approach to ensure both quantitative and qualitative data were used to inform Idaho’s food security research priorities. Figure 2 diagrams the quantitative and qualitative data tool development and data collection protocols employed. Figure 2 diagrams the quantitative and qualitative data tool development and data collection protocols employed. Survey data were analyzed via complete case analysis; pairwise deletion was employed to eliminate responses with missing values for variables being analyzed in each survey question. Descriptive statistics (frequency) for quantitative data were calculated using IBM SPSS Statistics for Windows, version 29 (IBM Corp., Armonk, NY, USA). Narrative analysis was employed to organize constructs and features emerging from the qualitative data. Utilizing content analysis, frequency of concepts and precipitating themes were manually compiled.

## 3. Results

### 3.1. Survey Findings

The survey was accessed by 212 individuals. After the removal of 15 records without responses, the analysis included 197 records. Using the 2023 US Census Data, a sample size of 385 respondents yielded a 95% confidence interval [18,19]. Though below the 385-participant threshold, the 13% (197/1575) response rate was within the acceptable range for a margin of error. The sampling process yielded a respondent pool representative of the state population breakdown by geographic disbursement and race/ethnicity [18]. A review of the distribution of missing survey data suggested that responses were missing completely at random; therefore, pairwise deletion calculations did not introduce the risk of bias [20]. Survey respondents were allowed to skip questions for any reason, which was likely a contributing factor.

Respondents were primarily White/Caucasian (155/197 [79%]) or Hispanic/Latino (18/197 [9%]). Combining living and working locales, respondents represented all 44 Idaho counties. The top three work settings were nonprofit associations (56/197 [28%]), higher education institutions (36/197 [18%]), and healthcare/social service agencies (31/187 [16%]). Nearly one-third of respondents (63/197 [32%]) were organizational leaders. Table 1 compares the population of Idaho with the purposeful sample and survey respondents.

**Figure 3:**
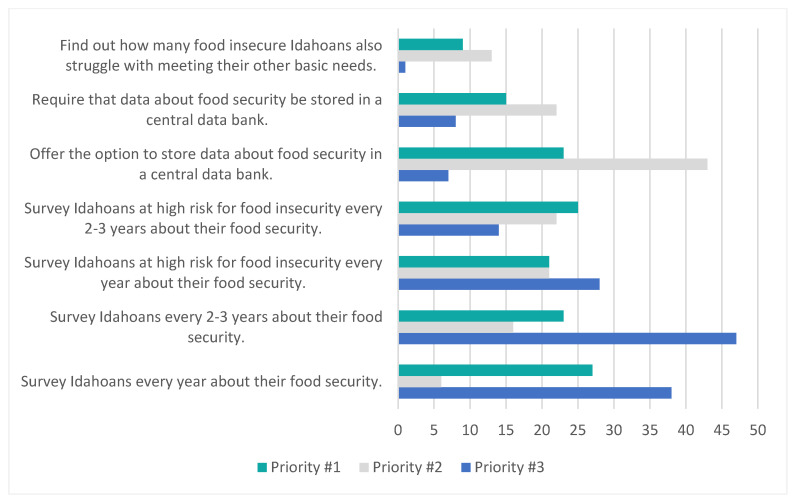
Prioritization of potential Idaho-wide food security screening practices.

High-ranking food security screening priorities included conducting annual food insecurity surveys (27/143 [19%] and developing an optional centralized databank for food insecurity datasets (43/143 [30%]). Over 60% of the respondents (107/172 [62%]) felt that Idaho schools should conduct food insecurity screens during the school year and summertime (see Figure 3). Eighty-three organizations (83/172 [48%]) collected data on food security; 44% (35/89) of those expressed willingness to share blinded datasets. Survey responses highlighted the need for innovative screening tools tailored to the experiences of Idahoans (105/169 [62%]) and easier to use in clinical and community settings (82/169 [49%]).

Respondents ranked American Indians (25/128 [20%]) and members of rural, migrant, and immigrant communities living in Idaho (24/128 [19%] at highest risk for food insecurity. Other high-risk groups (open response, n = 1) included the Asset Limited, Income Constrained, Employed (ALICE) population, children, college students, individuals living with chronic conditions, homeless, refugees, and seniors. Table 2 provides a breakdown of priority Idaho populations to include in food security studies.

**Figure 4:**
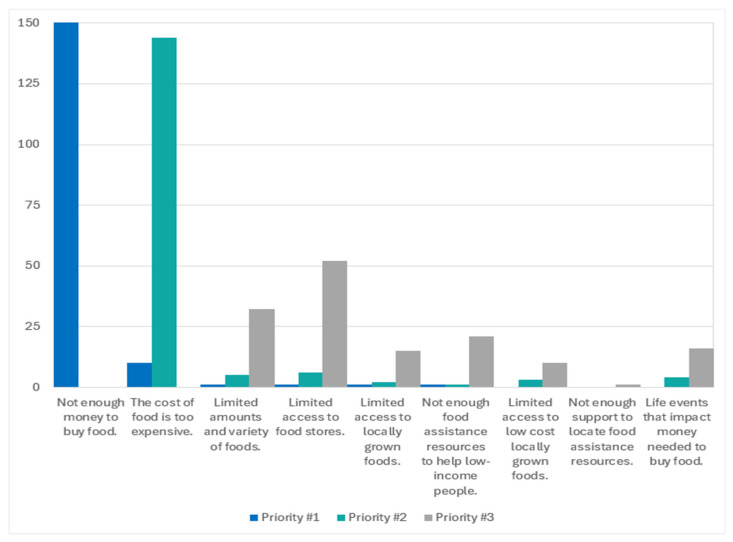
Ranking of social factors contributing to food insecurity among Idahoans.

The top-ranked contributory factors were insufficient funds for food, high food costs, and limited access to food stores (152/219, 91%; 144/166, 87%; 52/148, 35%, respectively). Additional contributing factors (open response, n = 1) were a lack of awareness about resources, no power for storing or cooking food, difficulty finding nutritious foods, and competing expenses (cost of living vs. wages). Figure 4 illustrates participant rankings of the social factors impacting food insecurity among Idahoans.

Over one-third (53/126 [42%]) ranked mental health as the top health concern associated with food insecurity. The negative impact on physical health ranked second, and overall quality of life third (47/126 [37%] and 44/126 [35%], respectively). Most respondents (143/169 [85%]) prioritized studying the impact of food insecurity, diet, and health among individuals living with chronic health issues (food sensitivities/allergies, diabetes, and disabling conditions). More than half (92/165 [56%]) rated conducting longitudinal studies on dietary patterns and health outcomes as a priority. Eighty-one percent (137/169) supported the need to evaluate the influence of local, state, and federal programs in mitigating the negative impacts of food insecurity.

Top research topics regarding local, state, and federal supplemental food programs were assessment of the efficacy of these programs in attenuating food insecurity (55/141 [39%]) and reducing food insecurity-related health risks (39/139 [28%]). The need for comparative effectiveness research on programs (local vs. state. vs. federal vs. combined funding) emerged (39/139 [28%]). Studies yielding a better understanding of improving access to programs were prioritized, as well as ones evaluating barriers to implementing local, state, and federal programs (95/147, 65%; 78/121, 65%; respectively). Considerations for addressing food insecurity included understanding the differences between rural and urban experiences. Pressing research needs (open-ended, n = 1) fell into six categories: researcher accountability, evidence, funding, food access, self-esteem, and programming. Researcher accountability included enhanced stakeholder collaboration and sharing of research findings. Evidence focused on equitable, data-driven solutions. Regarding funding, a state tax to help cover school lunch costs for all Idaho children was suggested. Food access included ensuring the adequacy of food supplies. Self-esteem meant evaluating programs that build self-efficacy to foster self-belief in one’s capability to reestablish economic stability. Programming suggestions called for strategies recognizing the disparate needs around the state. Table 3 details the most pressing needs for research on food security stratified by categorical topics.

### 3.2. Lunchtime Networking Assignment

The three emerging themes from this exercise were [community] programs working together better, rural vs. urban food security experience/needs, and program gaps/impact analyses. Most of the feedback collected fell into the latter theme (see Table 4).

### 3.3. Collaborative Agenda Development

**Figure 5:**
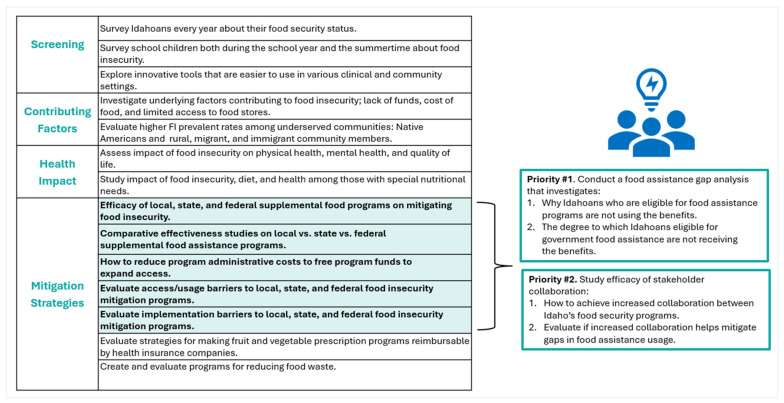
Top survey research topics mapped to the priorities created by Pillar 5 participants.

The in-person Pillar 5 session included 31 participants. This sample size reached saturation (strong external reliability = 12 to 13 participants) [21]. Though 85% (143/197) of survey respondents gave a high rating to study the impact of food insecurity, diet, and health among individuals with special nutritional needs, Pillar 5 participants prioritized food insecurity mitigation strategies (see Figure 5). This direction was guided by both the in-person stakeholder perspectives and experiences about the vital need to address the barriers to food security. It also drew on the survey finding that 81% (137/169) of respondents advocated for evaluation research on the efficacy of local, state, and federal supplemental food programs in mitigating the negative impacts of food insecurity.

Within food insecurity mitigation strategies, discussions focused on the efficacy of supplemental food programs, comparative effectiveness studies on food assistance programs, and investigation into strategies for reducing program administrative costs. Finally, studies on barriers to access/usage and the implementation of food insecurity mitigation programs were emphasized. Academic and non-academic stakeholders interacted as colleagues and peers during the prioritization exercise. The discussions included input from expert practitioners and gleaned insights from individuals with lived experiences. One example of a discussion theme impacted the final research priorities, including investigating a centralized system providing information about existing programs and services and offering an option to sign up for them through that system. As echoed in the survey results, the discussion highlighted a lack of awareness about many of the available programs, and, in Idaho, signing up can be difficult for those living in remote areas.

The first research objective prioritized by the Pillar 5 participants was to conduct food assistance gap analyses on (1) why Idahoans eligible for food assistance programs are not using those benefits, (2) how many eligible Idahoans are not receiving those benefits, and (3) gaps in urban and rural food assistance programming. Stakeholder collaboration was the second objective, specifically (1) investigating strategies to increase collaboration among Idaho’s food security programs and (2) evaluating if increased collaboration mitigates gaps in food assistance program usage.

### 3.4. CEnR Action Team

A Hunger Summit Pillar 5 action team was formed. The team meets regularly and provides quarterly updates to the IHRTF Executive Director and staff on the progress made toward achieving research agenda objectives. The team has presented our findings at multiple state-level conferences and events. Future, mixed-methodological research may include conducting focus groups with Idahoans participating in food assistance programs, additional statewide surveys, and studies of food security’s impact on health.

It was advantageous to include academics on the Pillar 5 team. They had grant writing, management, and data analysis skills, and access to resources (peer-reviewed literature, statistical tools, and individuals to help identify funding opportunities and assist with grant writing) that community members may be able to access. Research tasks have been incorporated into academic scholarship activities, and graduate students engaged in research projects, allowing community member engagement without overtasking. The incorporation of academics and student researchers ensures an asset-based framework for the research agenda.

## 4. Discussion

The population of Idaho spans suburban/urban and rural/remote regions with divergent socioeconomics, political ideologies, beliefs, and personal interests. Rural Idahoans are often labeled as having the fierce independence required to live in remote areas with limited resources [22,23]. Alm et al. found that Idaho’s southwest urban population center was more influential in policy decisions [24]. Also, of note, Idaho is one of 11 states in the USA that tax groceries; the Idaho tax rate is 6% of the food cost [25]. An actionable research agenda must thus be inclusive of the unique experiences and perspectives of the various regions of the state and ensure equitable representation among rural stakeholders; therefore, this intervention facilitated discussions among invested community stakeholders representing all 44 Idaho counties to establish state-specific research priorities.

Through a community–university partnership, stakeholders from across the state discussed food insecurity and how it impacts the well-being of Idahoans. The community engagement strategies included collecting baseline data via a statewide survey of community stakeholders and engaging participants of the 2023 IHRTF Hunger Summit in the prioritization and implementation process. The research design process ensured that all participants shared the same level of understanding regarding the insights gleaned from the survey findings. The survey enabled stakeholders across Idaho (n = 197, 44/44 counties) to participate in the advancement of the research agenda even if they were unable to attend the Summit. The in-person prioritization meeting facilitated a stakeholder-generated research agenda. Optimal community engagement was achieved, as degrees of engagement illustrated in the CEnR continuum were implemented.

The mixed-methods study design yielded comprehensive insights into the multifactorial dynamics influencing food security among Idahoans. The quantitative data offer an understanding of how stakeholders rank and prioritize food security issues. This analysis generated options for investigating the spectrum of factors contributing to the phenomenon. In contrast, qualitative data offer rich insights into the lived experience of hunger and pragmatic views into investigating why Idahoans are not leveraging existing resources aimed at improving food access issues. A decade ago, Sonnino et al. suggested that a food security research agenda must investigate social, economic, and environmental factors [26]. Of note, these factors map to the Idaho food security research agenda. For example, social factors such as knowledge about food assistance programs, access to reliable technology, or cultural and traditional values offer additional insights into potential investigations into gap analyses on why eligible Idahoans may not be receiving benefits. Potential economic factors of relevance to Idaho that might impact stakeholder collaboration include property rights issues impacting water access or food delivery routes, higher food prices in remote communities, and the length of food supply chains. Urban expansion and farm practices are environmental factors that may contribute to gaps in urban and rural food assistance programming.

Other studies documenting community engagement in developing food security research agendas (published in the peer-reviewed literature) are limited. Wentworth et al. reported on the experiences of five community-driven efforts to draft food systems research priorities [27]. Three communities included food security as a focal area—Ohio, New York, and Texas. The specific focus of the Ohio research topic and one of the New York topics were similar to the Idaho agenda in that they prioritized research studies on food access challenges. These two states, however, also ranked studies on food system transformation as high priorities for addressing food insecurity in their communities. The top Texas food security research item was to evaluate the efficacy of a program aimed at enhancing food security [27]. Similar program evaluations scored high in this study’s survey of Idahoans; respondents ranked as a top priority evaluating the efficacy of local, state, and federal supplemental food programs in mitigating food insecurity (55/141, 39.01%) and improving dietary patterns (41/141, 29.08%).

McCullum et al. convened 52 stakeholders (purposeful sample, upstate NY) to investigate how positions of power impact the development of a food security community-driven agenda setting [28]. The authors concluded that adherence to community-based participatory research processes allows for effective community engagement in food security agendas and overcomes the risk of individuals in power positions dominating agenda development. Of note, priority topics included food access challenges and self-esteem issues [28]. In the Idaho survey, helping “FI people get back on their feet and enhance their self-esteem” emerged as one of the most pressing issues; however, it was not selected as a research agenda item.

McElfish et al. reported on an academic health center employing the CEnR continuum to develop a research agenda to help address health disparities [8]. Thus, community stakeholders drove the prioritization process. Though research demonstrates a link between health equity and food security, food security did not emerge as a priority research topic in this agenda-setting exercise [8,29]. See Table 5 for a comparison of the Idaho research agenda with that of other states culled from peer-reviewed literature.

Nelson and Dodd advocated for the inclusion of graduate students in community-engaged research [30]. Lam et al. echoed this finding [31]. The graduate student research, completed as the initial project for the established research agenda, was also anchored by a cross-disciplinary university research organization and regular student–mentor meetings with the Pillar 5 team. The value of engaging emerging scholars in community–university partnerships within the food systems area emerged.

Strengths of this study include the participatory approach, which allowed individuals throughout the state to co-develop the research agenda. The mixed-methods approach facilitated both quantitative and qualitative data collection. Application of the CEnR continuum to the development of a statewide food security research agenda provides a framework that prescribes different types of data collection and encourages academics, practitioners, and participants with lived experience to form coalitions that can effectively carry out studies based on the research priorities identified. The framework and methodology used in this study can be replicated or adapted in other US states, as well as cities, counties, or other jurisdictions. There is also potential application for the development of other statewide research agendas in other areas of policy. The survey sample was sufficiently powered, enabling data collection from a diverse pool with targeted yet unique perspectives and experiences on the phenomenon of food security. The qualitative in-person participant group reached saturation. Recruitment approaches (purposeful plus snowball for the survey and convenience for the in-person session), however, introduced the risk of participant bias. The purposeful plus snowball sample has an additional risk of researcher bias, as many of the participants were selected by the researchers. In future studies, the generation of a random sample of stakeholders could help mitigate some of this risk of bias. Another limitation of the study was the failure to incorporate sex- and gender-based analyses (SGBA) into their research design; these data were not collected in the survey instrument or via the meeting registration process. Collection of SGBA variables in future efforts would offer additional insights parsed by demographics. Though it might appear that the survey sample lacked diversity, the race and ethnicity breakdown mirrors that of the Idaho population.

## 5. Conclusions

This study demonstrated a model for developing statewide research agendas and responding to national calls to action. The CEnR continuum proved efficacious for producing a stakeholder-generated research agenda. Leveraging a national strategic framework helped facilitate the development of a state food security agenda with actionable strategies for advancing the research within the proposed timeline. Capitalizing on an existing statewide gathering offered access to a range of community stakeholders. The value of engaging emerging scholars in public health research agenda development precipitated, especially regarding the goal for developing an asset-based research agenda.

The mixed-methods design coupled with the community engagement approach yielded realistic research priorities focused on addressing food access challenges among Idahoans. The first research objective was to conduct food assistance usage gap analyses; the second was to investigate barriers to collaboration among Idaho’s food security programs. Reflective of the 2022 Robert Wood Johnson report guidance on developing a state food security research agenda [6], a community-driven approach resulted in an agenda that embraces the strengths and resources of Idahoans. The focus on investigating the various food access challenges addresses the need to explore solutions for a state with diverse socioeconomics, political ideologies, beliefs, and personal interests [22,23].

The research topic categories of other USA states also included research topics on food systems transformation, evidence-based interventions, and self-esteem issues. Given that those categories were prioritized within the quantitative findings of this study, they may be future fodder for researchers searching to resolve the wicked problem of food insecurity in Idaho. Furthermore, study findings can be used to justify funding requests for research agenda topics, as well as additional areas of study.

## Figures and Tables

**Table 1:** Comparison of Idaho population, purposeful sample, and survey respondents.

Health District	State of Idaho ^1^		Purposeful Sample		Survey Respondents	
	No.	%	No.	%	No. ^1^	%
Central	569,107	29.32%	323	20.51%	150	29.76%
Eastern	257,974	13.29%	197	12.51%	54	10.71%
Panhandle	274,996	14.17%	206	13.08%	40	7.94%
North Central	114,035	5.88%	132	8.38%	50	9.92%
South Central	213,919	11.02%	211	13.40%	69	13.69%
Southeastern	184,005	9.48%	248	15.75%	67	13.29%
Southwest	326,956	16.84%	258	16.38%	74	14.68%
Total	1,940,992	100.00%	1575	100.00%	504	100.00%
Race/Ethnicity ^2^						
American Indian/Alaska Native	32,997	1.70%	--	--	4	2.03%
Asian	32,997	1.70%	--	--	1	0.51%
Black or African American	19,410	1.00%	--	--	1	0.51%
Hispanic or Latino	262,034	13.50%	--	--	18	9.14%
White or Caucasian	1,566,381	80.70%	--	--	155	78.68%
Two or more races	54,348	2.80%	--	--	--	--
Prefer not to answer	--	--	--	--	18	9.14%
Total	1,906,054	101.60%	--	--	197	100.01%
Organizational Affiliation						
Educational organization	--	--	205	13.02%	16	8.12%
Faith-based organization	--	--	165	10.48%	8	4.06%
Food industry company	--	--	44	2.79%	10	5.08%
Government agency	--	--	134	8.51%	34	17.26%
Healthcare/social service organization	--	--	359	22.79%	97	49.24%
Media group	--	--	12	0.76%	4	2.03%
Military	--	--	15	0.95%	2	1.02%
Nonprofit association	--	--	325	20.63%	19	9.64%
Tribal leadership	--	--	7	0.44%	3	1.52%
Other/not Known	--	--	309	19.62%	4	2.03%
Total	--	--	1576	100.00%	197	100.00%

Note: ^1^ Some respondents associated with multiple health districts. ^2^ Source: US Census Bureau. American Community Survey 5-Year Estimates.

**Table 2:** Idaho populations to include in studies on the phenomenon of food security.

	No.	%
Rural community members	24	16.22%
American Indians	25	16.89%
Migrant community members	24	16.22%
Recent immigrants	24	16.22%
Disabled individuals	15	10.14%
Socially disadvantaged individuals	16	10.81%
Other: Asset Limited, Income Constrained, Employed (ALICE) population, children, college students, individuals with chronic health issues, homeless, refugees, seniors	20	13.51%
Total responses	148	100.00%
No response/missing data	49	
Total	197	

**Table 3:** Most pressing needs for research on food security stratified by categorical topic.

Accountability of Researchers	Evidence-Based Investigations	Funding Opportunities	Food Access Challenges	Self-Esteem Issues
Build bridges to eliminate service gaps for FI Better stakeholder partnerships and communication Communicate the extent of the issue and share FI research findings Document success stories	Focus on equitable, evidence-based solutions Compare the ID FI phenomenon with experiences of other states Create a database of ID-specific model programs providing evidence of the impact of programs	More funding for food pantries Expand reach and increase the eligibility income cap for ID food assistance programs School lunch for all children (tax-based) School meal boxes for remote/rural community children	Eliminate barriers for Idahoans in need Reduce the stigma that healthy food is expensive and low-income people cannot afford to eat healthy Food is Medicine programs Explore why individuals do not utilize FI programs, which are prevalent in our community	A program is needed to help FI people get back on their feet and enhance their self-esteem Programs that encourage beneficiaries to give back more through volunteering and community service

FI = food insecurity, ID = Idaho.

**Table 4:** Emerging themes of lunchtime networking about Idaho food security research needs.

Programs Working Together Better What other public assistance programs or types of assistance are people, who are food insecure, on and how can we consolidate better to reduce costs and improve outcomes for everyone on the broad spectrum of those? Learning more about more programs, working together to find more tools. Agree with evaluating programs working together better The folks that I talked to during lunch all agreed that programs working together better would be really helpful at least for our program needs. When might we get major food corporations involved as far as the cost of food and why some people are not able to afford it. Similar thoughts on food producer involvement. Collaboration between all the nonprofit organizations, coming together on a regular basis [they might be able] to help close that gap and the populations they serve.
Rural vs. Urban Food Security Experience/Needs Rural vs. urban service needs, so we can understand what type of services are needed. Agree with evaluating rural vs. urban needs. Mine is like the rural vs. urban services. The rural vs. urban service needs because I’ve been a part of that, so I think that it’s really important. Causes of food insecurity: ○ If we ask the population that’s food insecure, what are the causes of food insecurity, what would that ranking look like? Is it primarily housing? Is it the primary cost of food? Is it primarily medical costs? ○ Looking more at the systemic structures that we live in and how they feed into access to food, holistically in our state. Local granular level data: ○ How to transition from federal and state level data to more local granular level data. I’m a land use planner, it’s very important for us to have a good sense for what’s happening in a neighborhood or census tract or block to see which population is underserved. They don’t have access to transportation to this grocery store. How can we get cities and municipalities to focus on creating their own local data? ○ The granular local data, we need more information on people that are really in it. Transportation factors: ○ How are we going to get those to grocery stores who don’t have transportation. ○ I really like the transportation idea because I know there’s a lot of people who get food stamps, but they don’t have the right transportation to get to places where they can get food.
Program Gaps/Impact Analyses Program gaps: ○ Are people getting services, is the information [about programs and services] getting out? ○ How do program policies map to the lived environment, how are people accessing services, who has access to services, where are services given, how we’re deciding that kind of thing? ○ There’s a big gap in people who are eligible for many of these services and programs and those receiving them. Who is not accessing them? I’m just very curious how to close that gap. Evaluate different populations [and their programs/services usage]. ○ The huge percentage of people who qualify but don’t access [programs/services], why don’t they access, and how do we help them access, so it may be a combination of the two. ○ Do we have the right people and their voices being heard, are we missing folks? Because if we’re missing the data from the people who we’re trying to serve, then we really can’t institute the right programs and everything else, so it goes hand in hand with them. Program impacts: ○ Looking at policies in this state and how they impact access to food, nutrition, physical activity, all those things that were listed on that one slide. ○ Are there cost savings to providing seniors with a home delivery meal? ○ What is the most viable way to get food insecure folks more money through policy? Is it a tax credit or trying to advocate for higher wages. What’s the best that we can do on an advocacy level? ○ For every dollar of food stamps, a dollar fifty comes out in the local economy. How does that stack up against other programs? ○ How do we get more resources to more people?

**Table 5:** Idaho food security research agenda vs. other state community-generated food security research topics culled from peer-reviewed literature.

Author	State	Focus Area	Food Security Research Gap
Gordon et al.	Idaho (Statewide)	Food Access Challenges	Conduct food assistance gap analyses on (1) why Idahoans eligible for food assistance programs are not using those benefits, (2) how many eligible Idahoans are not receiving those benefits, and (3) gaps in urban and rural food assistance programming. Investigate strategies to increase collaboration among Idaho’s food security programs and evaluate if increased collaboration mitigates gaps in food assistance program usage.
Wentworth et al. [27]	Ohio (Cleveland)	Food Systems Transformation	Evaluate… “how to approach food systems transformation from a systems perspective.”
		Food Access Challenges	“Identify necessary conditions and connections in the system in a way that promotes equity in food access and nutrition.”
Wentworth et al. [27]	New York (Albany)	Food Access Challenges	“Quantify environmental, nutritional, and health impacts of the food recovery and redistribution system… and ascertain impacts under proposed policy scenarios.”
McCullum et al. [28]	New York (upstate county)	Food Systems Transformation	Investigate “new agriculture, to explore agricultural economic opportunities.”
		Food Access Challenges	How to improve the “distribution of surplus food, to strengthen a distribution network for surplus and leftover food; … raise awareness of consumers as to the food and fiber system and related life skills through education; food processing and marketing, to give … producers outlets for their products;
		Self-Esteem Issues	How to enhance “family and community values, to encourage and nurture a commitment to personal responsibility and wider relationships through building community.”
Wentworth et al. [27]	Texas (Austin)	Evidence-Based Investigations	“Empirically assess the impact of the Fresh For Less (FFL) initiative on individual food security and vegetable intake. FFL involved the placement of non-traditional food retail locations (e.g., healthy corners stores, farm stands, mobile markets) in low-income, ethnically and racially diverse communities.” “[Analyze] … if/how community members utilize new retail options, if new retail options reach the intended audience, and impact dietary behavior.”

## Data Availability

The original contributions presented in this study are included in the article/Supplementary Material. Further inquiries can be directed to the corresponding author.

## Supplementary Materials

The following supporting information can be downloaded at: https://www.mdpi.com/article/10.3390/ijerph21121677/s1, Table S1: Good Reporting of A Mixed Methods Study (GRAMMS); Figure S1: Food Security Research Needs Survey Tool; Table S2: Comparison of Idaho Population, Purposeful Sample, and Survey Respondents; Table S3: Research on Screening for Food Security; Table S4: Research on Factors Contributing to Food Insecurity; Table S5: Research on the Impact of FI on Dietary Choices and Associated Health Outcomes; Table S6: Research on Local, State, and Federal Strategies to Address Food Insecurity.
