# Supplementary material for: Utilization of Participatory Research Theory and a National Framework to Advance a State Food Security Research Agenda: A Mixed-Methods Study
Source: Int J Environ Res Public Health. 2024 Dec 17;21(12):1677. doi: 10.3390/ijerph21121677 (PMC11675214; doi:10.3390/ijerph21121677)
Supplement: Supplementary file 1 [file ijerph-21-01677-s001.zip › ijerph-3325266-supplementary.pdf]

Supplemental Materials

Table S1. Good Reporting of A Mixed Methods Study (GRAMMS)

Best Practice	Manuscript Page(s)
1. Describe the justification for using a mixed methods approach to the research question	Page 2-3
2. Describe the design in terms of:	
• Purpose	Pages 2-3
• Priority	Page 2-3
• Sequence of methods	Pages 2-3
3. Describe each method in terms of:	
• Sampling	Page 3
• Data collection	Pages 3
• Analysis	Pages 3-7
4. Describe where the integration:	
• Has occurred	Pages 3-7
• How it has occurred	Pages 3-7
• Who has participated in it	Pages 3-7
5. Describe any limitation of one method associated with the presence of the other method	Pages 7-8
6. Describe any insights gained from mixing or integrating methods	Pages 7-8

Source: O'Cathain A, Murphy E, Nicholl J. The quality of mixed methods studies in health services research. J Health Serv Res Policy. 2008;13(2):92-98.

Figure S1. Food Security Research Needs Survey Tool

DEMOGRAPHICS

Q1. Please tell us a little about yourself. Select the Idaho county that you live in:

- I don't live in Idaho (1)
- Ada (2)
- Adams (3)
- Bannock (4)
- Bear Lake (5)
- Benewah (6)
- Bingham (7)
- Blaine (8)
- Boise (9)
- Bonner (10)
- Bonneville (11)
- Boundary (12)
- Butte (13)
- Camas (14)
- Cassia (15)
- Canyon (16)
- Caribou (17)
- Clark (18)
- Clearwater (19)
- Custer (20)
- Elmore (21)
- Franklin (22)
- Fremont (23)
- Gem (24)
- Gooding (25)
- Idaho (26)
- Jefferson (27)
- Jerome (28)
- Kootenai (29)
- Latah (30)
- Lemhi (31)
- Lewis (32)
- Lincoln (33)
- Madison (34)
- Minidoka (35)
- Nez Perce (36)
- Oneida (37)
- Owyhee (38)
- Payette (39)
- Power (40)
- Shoshone (41)
- Teton (42)
- Twin Falls (43)
- Valley (44)
- Washington (45)

Q2. Select the Idaho county or counties that you work in (select all that apply):

- I work on a national level (1)
- I work regionally, including ID (2)
- I am currently not employed (3)
- Ada (4)
- Adams (5)
- Bannock (6)
- Bear Lake (7)
- Benewah (8)
- Bingham (9)
- Blaine (10)
- Boise (11)
- Bonner (12)
- Bonneville (13)
- Boundary (14)
- Butte (15)
- Camas (16)
- Cassia (17)
- Canyon (18)
- Caribou (19)
- Clark (20)
- Clearwater (21)
- Custer (22)
- Elmore (23)
- Franklin (24)
- Fremont (25)
- Gem (26)
- Gooding (27)
- Idaho (28)
- Jefferson (29)
- Jerome (30)
- Kootenai (31)
- Latah (32)
- Lemhi (33)
- Lewis (34)
- Lincoln (35)
- Madison (36)
- Minidoka (37)
- Nez Perce (38)
- Oneida (39)
- Owyhee (40)
- Payette (41)
- Power (42)
- Shoshone (43)
- Teton (44)
- Twin Falls (45)
- Valley (46)
- Washington (47)

Q3. Select the answer that best describes the type of organization that you work at (select all that apply):
- Faith-based organization (1)
- Food industry company (2)
- Government agency (3)
- Health insurance organization (4)
- Healthcare/social service organization (5)
- Higher education (college/university) (6)
- Secondary education (elementary/middle/high school) (7)
- Media group (8)
- Military agency (9)
- Nonprofit association (10)
- Tribal Leadership Council (11)
- Other (please describe) (12) ____

Q4. Select the answer that best describes the function that you perform at work (select all that apply):
- Teacher/faculty (1)
- Researcher (2)
- Healthcare provider (3)
- Social services provider (4)
- Organizational leader/administrator (5)
- Organizational staff person (6)
- Policy analysis (7)
- Religious leader/staff (8)
- Volunteer (9)
- Other (please describe) (10) ____

Q5. Select the answer that best describes your race/ethnicity:
- American Indian (2)
- Asian (2)

- o Black or African American (3)
- o Hispanic/Latino (4)
- o Native Hawaiian (5)
- o White or Caucasian (6)
- o Other (please describe) (7) ____
- o Prefer not to answer (8)

Q6. Select your age range:
- o 18 to 28 years (1)
- o 29 to 49 years (2)
- o 49 to 69 years (3)
- o 70 years or older (4)
- o Prefer not to answer (5)

RESEARCH ON SCREENING FOR FOOD INSECURITY

Please let us know your thoughts on how to better understand food security among Idahoans. We want to learn your thoughts about what research needs to be done on this topic. Before you complete the survey, take a minute to review the following US Department of Agriculture definitions for food security and food insecurity:

Food Security
- High food security: No reported indications of food-access problems or limitations.
- Marginal food security: One or two reported indications—typically of anxiety over food sufficiency or shortage of food in the house. Little or no indication of changes in diets or food intake.

Food Insecurity
- Low food security: Reports of reduced quality, variety, or desirability of diet. Little or no indication of reduced food intake.
- Very low food security: Reports of multiple indications of disrupted eating patterns and reduced food intake.

Q7. Which research activity do you think is most important for understanding food security among Idahoans? Please rank the following research concepts in order of importance. Start with the most important at the top of the list and end with the least important at the bottom of the list.
- o Survey Idahoans every year about their food security. (1)
- o Survey Idahoans every two or three years about their food security. (2)
- o Survey Idahoans at high risk for food insecurity every year about their food security. (3)
- o Survey Idahoans at high risk for food insecurity every two or three years about their food security. (4)
- o Offer the option to store data about food security in a central data bank. (5)
- o Require that data about food security be stored in a central data bank. (6)
- o Find out how many food insecure Idahoans also struggle with meeting their other basic needs (such as housing). (7)

Q8. In your opinion, should Idaho schools be required to collect data about how many students are food insecure?
- o Yes, but only during the school year (1)
- o Yes, but only during the summer (2)
- o Yes, both during the school year and the summertime (3)
- o Other (please describe) (4) ____
- o No (5)
- o Unsure (6)

Q9. Does your organization/community collect information about food security among Idahoans?
- o No (1)
- o Yes (2)
- o Unsure (3)

Display these questions if Q9 = Yes

Q9a. My organization/community (select all that apply):
- o Screens for food insecurity and records the information in an electronic health record, student file, etc. (1)
- o Surveys stakeholders and gathers findings about food insecurity status among patrons (2)
- o Documents the numbers and types of referrals to supplemental food programs and similar social services (3)
- o Conducts research studies on food insecurity-related topics (4)
- o Collects data for policy position papers (5)
- o Other (please describe) (6) ____

Q9b. Are you willing to share your food security data?
- o Yes (1)
- o Yes, but… (please describe conditions for sharing your data) (2) _______________
- o No (3)
- o Unsure (4)
- o Other (please describe) (5) ____

Q10. The federal government provides free tools for surveying groups about food security. Do you think more research is needed to assess how well those tools apply to the experiences of Idahoans?

- o Yes (1)
- o Yes, and more research is also needed on innovative tools that are easier to use in various clinical and community settings (2)
- o No (3)
- o No, but better availability of the existing tools is needed (4)
- o Unsure (5)

RESEARCH ON FACTORS CONTRIBUTING TO FOOD INSECURITY

Q11. What social factors do you think contribute to food insecurity in Idaho (select all that apply)?

- o Not enough money to buy food (1)
- o The cost of food is too expensive (2)
- o Limited amounts and variety of foods (3)
- o Limited access to food stores (4)
- o Limited access to locally grown foods (5)
- o Not enough food assistance resources to help low-income people (6)
- o Limited access to low-cost locally grown foods (7)
- o Not enough support to locate food assistance resources (8)
- o Life events that impact money needed to buy food (e.g., holiday purchases, tax time, etc.) (9)
- o Other (please describe) (10) ____

Q12. Do you feel there are other food security issues affecting Idahoans that are not well understood or less known?

____

Q13. Do you feel it is important to evaluate local, state, and federal supplemental food programs for their ability to improve potential negative impacts of food insecurity (such as physical/mental/social health, quality of life, educational level, sustained employment, etc.)?

- o Yes (1)
- o No (2)
- o Unsure (3)

Display this question if Q12 = Yes

Q13a. Please rank the following potential negative impacts of food insecurity by order of importance.
Start with the most important potential negative impact of food insecurity at the top of the list and end with the least important at the bottom of the list.

______ Physical health (1)
______ Mental health (2)
______ Social health (3)
______ Quality of life (4)
______ Educational level (5)
______ Sustained employment (6)
______ Other (please describe; leave blank if no response) (7)

RESEARCH ON THE IMPACT OF FI ON DIETARY CHOICES AND ASSOCIATED HEALTH OUTCOMES

Q14. Do you feel it is important to study the impact of food insecurity, diet, and health among individuals with special nutritional needs?

- o Yes (1)
- o No (2)
- o Unsure (3)

Display this question if Q14 = Yes

Q14a Since you feel it is important to study the impact of food insecurity, diet, and health among individuals with nutritional needs, please rank the following individuals by order of who should be studied. Start with the individuals who you feel should be studied most, and end with those you feel should be studied least.

______ Rural community members (1)
______ American Indians (2)
______ Migrant community members (3)
______ Recent immigrants (4)
______ Disabled individuals (5)
______ Socially disadvantaged individuals (6)
______ Other (please describe; leave blank if no response) (7)

Q15. Longitudinal studies track impacts across long amounts of time. As a result, these studies cost a lot. However, because of their length, they also provide valuable insights. Do you think there is a need for longitudinal studies on dietary patterns among food insecure Idahoans and health outcomes throughout life stages?

- o Yes (1)
- o Yes, but... (please describe) (2) ____
- o No (3)
- o Unsure (4)

RESEARCH ON LOCAL, STATE, AND FEDERAL STRATEGIES TO ADDRESS FI

Q16. Please rank the following food security research topics by order of importance. Start with the most important research topic at the top and end with the least important topic at the bottom.

- o How well local, state, and federal supplemental food programs address food insecurity among Idahoans (1)
- o How well local, state, and federal supplemental food programs improve eating patterns among food insecure Idahoans (2)
- o How well supplemental food programs combat the potential health risks associated with food insecurity among Idahoans (3)
- o Comparison of the effectiveness of local vs. state vs. federal vs. combined funding of programs addressing food insecurity in ID (4)
- o The degree to which supplemental food programs break the cycle of food insecurity across generations (5)
- o Studies to help inform nutrition and food security policy (6)
- o Other (please describe; leave blank if no response) (7)

Q17. Which of the following food security topics do you think need further study? Select all that apply.

- o How to improve access to and use of state, local, and federal assistance programs by reducing the management steps required to run those programs (1)
- o Evaluate barriers for individuals to access and use local, state, and federal programs aimed at addressing food insecurity among Idahoans (2)
- o Evaluate barriers to implementing local, state, and federal programs aimed at addressing food insecurity among Idahoans (3)
- o Studies that justify ongoing funding by local, state, and federal agencies for programs aimed at addressing food insecurity among Idahoans (4)
- o Other (please describe) (5) ____

Q18. Which three topics do you think need further study to ensure no Idahoans go hungry? Select three.

- o Better access to food via fruit and vegetable prescription programs paid for by health insurance programs (1)
- o Better access to SNAP (food stamps) and other income-based supplemental food programs (2)
- o Ongoing funding for rural food cooperatives to increase access for those living in food deserts (3)
- o Providing funds to encourage grocery stores to increase access to healthy, affordable food in communities that currently lack these options (4)
- o Studying climate-smart agricultural innovations (5)
- o Developing crops and livestock that are more genetically resilient (6)
- o Creating programs that reduce food waste (7)
- o Other (please describe) (8) ____

Q19. Please tell us what you think are the most pressing needs for research on food security. Feel free to include any final thoughts as well.

____

Table S2. Comparison of Idaho Population, Purposeful Sample, and Survey Respondents

Health District	Idaho County	State of Idaho[1]		Purposeful Sample		Survey Respondents	
		No.	%	No.	%	No.	%
Central	Ada	518,907	26.73%	244	15.49%	134	26.59%
	Boise	8,333	0.43%	18	1.14%	3	0.60%
	Elmore	29,403	1.51%	33	2.10%	6	1.19%
	Valley	12,464	0.64%	28	1.78%	7	1.39%
	Subtotal	569,107	**29.32%**	323	20.51%	150	**29.76%**
Eastern	Bonneville	129,496	6.67%	61	3.87%	16	3.17%
	Clark	806	0.04%	6	0.38%	3	0.60%
	Custer	4,506	0.23%	12	0.76%	2	0.40%
	Fremont	13,978	0.72%	22	1.40%	4	0.79%
	Jefferson	33,428	1.72%	20	1.27%	12	2.38%
	Lemhi	8,240	0.42%	7	0.44%	3	0.60%
	Madison	54,976	2.83%	38	2.41%	7	1.39%
	Teton	12,544	0.65%	31	1.97%	7	1.39%
	Subtotal	257,974	**13.29%**	197	12.51%	54	**10.71%**
Panhandle	Benewah	10,370	0.53%	35	2.22%	8	1.59%
	Bonner	54,414	2.80%	34	2.16%	7	1.39%
	Boundary	13,345	0.69%	32	2.03%	6	1.19%
	Kootenai	183,578	9.46%	75	4.76%	13	2.58%
	Shoshone	13,289	0.68%	30	1.90%	6	1.19%
	Subtotal	274,996	**14.17%**	206	13.08%	40	**7.94%**
North Central	Clearwater	9,015	0.46%	10	0.63%	3	0.60%
	Idaho	17,592	0.91%	24	1.52%	12	2.38%
	Latah	40,978	2.11%	37	2.35%	19	3.77%
	Lewis	3,446	0.18%	13	0.83%	7	1.39%
	Nez Perce	43,004	2.22%	48	3.05%	9	1.79%
	Subtotal	114,035	**5.88%**	132	8.38%	50	**9.92%**
South Central	Blaine	24,866	1.28%	33	2.10%	11	2.18%
	Camas	1,153	0.06%	18	1.14%	2	0.40%
	Cassia	25,655	1.32%	32	2.03%	6	1.19%
	Gooding	15,715	0.81%	16	1.02%	9	1.79%
	Jerome	25,311	1.30%	18	1.14%	10	1.98%
	Lincoln	5,329	0.27%	18	1.14%	5	0.99%
	Minidoka	22,194	1.14%	21	1.33%	6	1.19%
	Twin Falls	93,696	4.83%	55	3.49%	20	3.97%
	Subtotal	213,919	**11.02%**	211	13.40%	69	**13.69%**
Southeastern	Bannock	89,517	4.61%	91	5.78%	32	6.35%
	Bear Lake	6,722	0.35%	18	1.14%	5	0.99%
	Bingham	49,923	2.57%	40	2.54%	13	2.58%
	Butte	2,684	0.14%	15	0.95%	3	0.60%
	Caribou	7,190	0.37%	14	0.89%	4	0.79%
	Franklin	15,189	0.78%	22	1.40%	3	0.60%
	Oneida	4,712	0.24%	23	1.46%	2	0.40%
	Power	8,068	0.42%	25	1.59%	5	0.99%
	Subtotal	184,005	**9.48%**	248	15.75%	67	**13.29%**
Southwest	Adams	4,817	0.25%	16	1.02%	2	0.40%
	Canyon	251,065	12.93%	174	11.05%	36	7.14%
	Gem	20,418	1.05%	26	1.65%	4	0.79%
	Owyhee	12,613	0.65%	10	0.63%	5	0.99%
	Payette	26,956	1.39%	21	1.33%	1	0.20%
	Washington	11,087	0.57%	11	0.70%	26	5.16%
	Subtotal	326,956	**16.84%**	258	16.38%	74	**14.68%**
	Total	1,940,992	100.00%	1,575	100.00%	504	100.00%
**Race/Ethnicity[1]**							
American Indian/Alaska Native		32,997	1.70%	--	--	4	2.03%
Asian		32,997	1.70%	--	--	1	0.51%
Black or African American		19,410	1.00%	--	--	1	0.51%
Hispanic or Latino		262,034	13.50%	--	--	18	9.14%
White or Caucasian		1,566,381	80.70%	--	--	155	78.68%
Two or more races		54,348	2.80%	--	--	--	--
Prefer not to answer		--	--	--	--	18	9.14%
Total		1,906,054	101.60%	--	--	197	100.01%

Note: 1. Source: US Census Bureau. American Community Survey 5-Year Estimates.

Table S3. Research on Screening for Food Security

Most Important Research Activities for Understanding Food Security Among Idahoans						
	No.	%	No.	%	No.	%
	Priority #1		*Priority #2*		*Priority #3*	
Survey Idahoans every year about their food security.	27	18.88%	6	4.20%	38	26.57%
Survey Idahoans every 2-3 years about their food security.	23	16.08%	16	11.19%	47	32.87%
Survey Idahoans at high risk for food insecurity every year about their food security.	21	14.69%	21	14.69%	28	19.58%
Survey Idahoans at high risk for food insecurity every 2-3 years about their food security.	25	17.48%	22	15.38%	14	9.79%
Offer the option to store data about food security in a central data bank.	23	16.08%	43	30.07%	7	4.90%
Require that data about food security be stored in a central data bank.	15	10.49%	22	15.38%	8	5.59%
Find out how many food insecure Idahoans also struggle with meeting their other basic needs.	9	6.29%	13	9.09%	1	0.70%
Total Responses	143	100.00%	143	100.00%	143	100.00%
No Response/Missing Data	54		54		54	
Total	197		197		197	

Idaho Schools Should be Required to Collect Data about How Many Students Are Food Insecure		
Yes, but only during the school year.	20	11.63%
Yes, both during the school year and the summertime.	107	62.21%
Other<br>• Offer information to all families describing resources in the area.<br>• Provide voluntary data collection; ask families but not require a response.	8	4.65%
No.	20	11.63%
Unsure.	17	9.88%
Total Responses	172	100.00%
No Response/Missing Data	25	
Total	197	

Organization/Community Collects Information about Food Security among Idahoans		
No	56	32.56%
Yes	83	48.26%
Unsure	33	19.19%
Total Responses	172	100.00%
No Response/Missing Data	25	
Total	197	

If Collect Food Security Data, Types of Activities (n=83)		
Screen for food insecurity and record information in electronic health records, student files, etc.	32	16.58%
Survey stakeholders and gather findings about food insecurity status among patrons.	20	10.36%
Document the numbers/types of referrals to supplemental food programs/social services.	19	9.84%
Conduct research studies on food insecurity-related topics.	12	6.22%
Collect data for policy position paper.	7	3.63%
Other<br>• Report food insecurity prevalence statistics to the Social Security Administration.<br>• Collect household income and SNAP data for all children enrolled in care at participating childcare and afterschool sites.<br>• Report to Idaho Food Bank and El-Ada Community Action Partnership.<br>• Collect food security prevalence via the College Health Survey.	14	7.25%
Total Responses (check all that apply)	193	100.00%
No Response/Missing Data	5	
Total	78	

If Collect Food Security Data, Willing to Share Blinded Set (n=83)		
Yes.	19	23.75%
Yes, but… (please describe conditions for sharing your data).	16	20.00%
No.	3	3.75%
Unsure.	35	43.75%
Other.<br>• Requires organizational approval.<br>• Must contain no personally identifiable information.<br>• Aggregate only.<br>• Only for the organization that sponsored the data collection activity.	7	8.75%
Total Responses	80	100.00%
No Response/Missing Data	3	
Total	83	

More Efficacy Testing of Free Federal Government Food Security Screening Tools		
Yes.	23	13.61%
Yes, and research innovative tools that are easier to use in clinical and community settings.	82	48.52%
No.	9	5.33%
No, but better availability of the existing tools is needed.	16	9.47%
Unsure.	39	23.08%
Total Responses	169	100.00%
No Response/Missing Data	28	
Total	197	

Table S4. Research on Factors Contributing to Food Insecurity

Social Factors that Contribute to Food Insecurity in Idaho

	No.	%	No.	%	No.	%
	Priority #1		*Priority #2*		*Priority #3*	
Not enough money to buy food.	153	90.53%	0	0.00%	0	0.00%
The cost of food is too expensive.	10	5.92%	144	86.75%	0	0.00%
Limited amounts and variety of foods.	1	0.59%	5	3.01%	32	21.62%
Limited access to food stores.	1	0.59%	6	3.61%	52	35.14%
Limited access to locally grown foods.	1	0.59%	2	1.20%	15	10.14%
Not enough food assistance resources to help low-income people.	1	0.59%	1	0.60%	21	14.19%
Limited access to low cost locally grown foods.	0	0.00%	3	1.81%	10	6.76%
Not enough support to locate food assistance resources.	0	0.00%	0	0.00%	1	0.68%
Life events that impact money needed to buy food.	0	0.00%	4	2.41%	16	10.81%
Other<br>• Lack of local jobs not requiring a 75 to 100-mile daily commute, low wages.<br>• Systemic issues that cause poverty and access restrictions (transportation challenges, inflated cost of living, skewed median income levels).<br>• Lack of education/skills (food prep, life skills).<br>• Stigma, refusing assistance due to pride or shame.	2	1.18%	1	0.60%	1	0.68%
Total Responses	169	100.00%	166	100.00%	148	100.00%
No Response/Missing Data	28		31		49	
Total	197		197		197	

Other Food Security Issues Affecting Idahoans that are not Well Understood or Less Known

- Resources and services designed for more populated areas don't work in rural Idaho.
- Issues specific to tribal nations—impacts of diminishing water supply, rising transportation and fuel costs.
- Webs of complexity involving social, economic, and environmental factors.
- Gaps/changes in food assistance program edibility thresholds.
- Reluctance of those who are food insecure to do a screening assessment.
- Effects of social pressures such as racism and sexism.

Evaluate the Efficacy of Local, State, and Federal Supplemental Food Programs in Mitigating Negative Impacts of Food Insecurity

Yes	137	81.07%
No	8	4.73%
Unsure	24	14.20%
Total Responses	169	100.00%
No Response/Missing Data	28	
Total	197	

Potential Negative Impacts of Food Insecurity

Physical health	*Priority #1*		*Priority #2*		*Priority #3*	
Mental health	47	37.30%	17	13.49%	3	2.38%
Social health	43	34.13%	53	42.06%	1	0.79%
Quality of life	22	17.46%	40	31.75%	18	14.29%
Educational level	8	6.35%	10	7.94%	44	34.92%
Sustained employment	5	3.97%	4	3.17%	31	24.60%
Other<br>• They are all essential impacts to study.<br>• Poor dental health.<br>• Generational impacts.<br>• Adds additional challenges to maintaining sobriety.	1	0.79%	2	1.59%	26	20.63%
Total Responses (select all that apply)	126	100.00%	126	100.00%	126	100.00%
No Response/Missing Data	71		71		71	
Total	197		197		197	

Table S5. Research on the Impact of FI on Dietary Choices and Associated Health Outcomes

**Important to Study the Impact of Food Insecurity, Diet, and Health among Individuals with Special Nutritional Needs**		
	**No.**	**%**
Yes	143	84.62%
No	7	4.14%
Unsure	19	11.24%
Total Responses	169	100.00%
No Response/Missing Data	28	
Total	197	
**Evaluate Phenomenon of Food Insecurity among Idaho Populations**		
Rural community members	24	16.22%
American Indians	25	16.89%
Migrant community members	24	16.22%
Recent immigrants	24	16.22%
Disabled individuals	15	10.14%
Socially disadvantaged individuals	16	10.81%
Other<br>• All groups are equally important.<br>• Survey to uncover the most food insecure group.<br>• Refugees.<br>• Asset Limited, Income Constrained, Employed (ALICE) population.<br>• Individuals with chronic health issues (food sensitivities/allergies, diabetes, disabling conditions).<br>• College students.<br>• Seniors.	20	13.51%
Total Responses	148	100.00%
No Response/Missing Data	49	
Total	197	
**Organization/Community Collects Information about Food Security among Idahoans**		
No	56	32.56%
Yes	83	48.26%
Unsure	33	19.19%
Total Responses	172	100.00%
No Response/Missing Data	25	
Total	197	
**Conduct Longitudinal Studies on Dietary Patterns among Food Insecure Idahoans and Health Outcomes across the Life Cycle**		
Yes	92	55.76%
Yes, but	14	8.48%
No	24	14.55%
Unsure	35	21.21%
Total Responses	165	100.00%
No Response/Missing Data	32	
Total	197	

Table S6. Research on Local, State, and Federal Strategies to Address Food Insecurity

**Food Security Research Topics Ranked in Order of Priority**						
	**No.**	**%**	**No.**	**%**	**No.**	**%**
	Priority #1		*Priority #2*		*Priority #3*	
Efficacy of local, state, and federal supplemental food programs in mitigating food insecurity.	55	39.01%	9	6.47%	20	14.39%
Efficacy of local, state, and federal supplemental food programs in improving dietary patterns.	41	29.08%	19	13.67%	27	19.42%
Efficacy of supplemental food programs in combating food insecurity-related health risks.	20	14.18%	39	28.06%	30	21.58%
Comparative effectiveness studies of food insecurity programs.	13	9.22%	31	22.30%	39	28.06%
Efficacy of supplemental food programs in breaking the generational cycle of food insecurity.	9	6.38%	28	20.14%	15	10.79%
Studies to help inform nutrition and food security policy.	3	2.13%	12	8.63%	8	5.76%
Other<br>• All, too difficult to rank.<br>• Focus on eligibility, participation, barriers, disincentives, etc. of supplemental food programs.<br>• Deficiencies in the market food system that have led to the need for supplemental food programs.	0	0.00%	1	0.72%	0	0.00%
Total Responses	141	100.00%	139	100.00%	139	100.00%
No Response/Missing Data	56		58		58	
Total	197		197		197	
**Food Security Research Topics Requiring Further Study**						

How to improve access to and use of state, local, and federal assistance programs by reducing the management steps required to run those programs.	95	64.63%	0	0.00%	0	0.00%
Evaluate barriers for individuals to access and use local, state, and federal programs aimed at addressing food insecurity among Idahoans.	40	27.21%	78	64.46%	0	0.00%
Evaluate barriers to implementing local, state, and federal programs aimed at addressing food insecurity among Idahoans.	1	0.68%	29	23.97%	65	78.31%
Studies that justify ongoing funding by local, state, and federal agencies for programs aimed at addressing food insecurity among Idahoans.	7	4.76%	12	9.92%	18	21.69%
Other<br>• How to omit barriers and make food accessible for all.<br>• Studies that directly result in ending food insecurity.	4	2.72%	2	1.65%	0	0.00%
Total Responses (select all that apply)	147	100.00%	121	100.00%	83	100.00%
No Response/Missing Data	50		76		114	
Total	197		197		197	

Three Topics Requiring Further Study

	Topic #1		*Topic #2*		*Topic #3*	
Health insurance coverage for fruit and vegetable prescription programs.	94	60.65%	0	0.00%	0	0.00%
Better access to SNAP (food stamps) and other income-based supplemental food programs.	37	23.87%	51	35.17%	0	0.00%
Ongoing funding for rural food cooperatives to increase access for those living in food deserts.	17	10.97%	48	33.10%	24	19.05%
Providing funds to encourage grocery stores to increase access to healthy, affordable food in communities that currently lack these options	2	1.29%	24	16.55%	36	28.57%
Studying climate-smart agricultural innovations.	1	0.65%	11	7.59%	6	4.76%
Developing crops and livestock that are more genetically resilient.	0	0.00%	1	0.69%	3	2.38%
Creating programs that reduce food waste.	0	0.00%	10	6.90%	52	41.27%
Other<br>• Cost of living studies.<br>• Strategies for eliminating hunger—basic income programs.<br>• Evaluation of the economic impact of lower income thresholds for SNAP.	4	2.58%	0	0.00%	5	3.97%
Total Responses	155	100.00%	145	100.00%	126	100.00%
No Response/Missing Data	42		52		71	
Total	197		197		197	
